# Inhibition of RelA-Ser536 Phosphorylation by a Competing Peptide Reduces Mouse Liver Fibrosis Without Blocking the Innate Immune Response

**DOI:** 10.1002/hep.26068

**Published:** 2013-01-08

**Authors:** Anna Moles, Ana M Sanchez, Paul S Banks, Lindsay B Murphy, Saimir Luli, Lee Borthwick, Andrew Fisher, Steven O’Reilly, Jacob M van Laar, Steven A White, Neil D Perkins, Alastair D Burt, Derek A Mann, Fiona Oakley

**Affiliations:** 1 Fibrosis Research Group, Institute of Cellular Medicine, Newcastle UniversityNewcastle upon Tyne, United Kingdom; 2 Institute for Cell and Molecular Biosciences, Newcastle UniversityNewcastle upon Tyne, United Kingdom; 3 Musculoskeletal Research Group, Institute of Cellular Medicine, Newcastle UniversityNewcastle upon Tyne, United Kingdom

## Abstract

Phosphorylation of the RelA subunit at serine 536 (RelA-P-Ser536) is important for hepatic myofibroblast survival and is mechanistically implicated in liver fibrosis. Here, we show that a cell-permeable competing peptide (P6) functions as a specific targeted inhibitor of RelA-P-Ser536 *in vivo* and exerts an antifibrogenic effect in two progressive liver disease models, but does not impair hepatic inflammation or innate immune responses after lipopolysaccharide challenge. Using kinase assays and western blotting, we confirm that P6 is a substrate for the inhibitory kappa B kinases (IKKs), IKKα and IKKβ, and, in human hepatic myofibroblasts, P6 prevents RelA-P-Ser536, but does not affect IKK activation of IκBα. We demonstrate that RelA-P-Ser536 is a feature of human lung and skin fibroblasts, but not lung epithelial cells, *in vitro* and is present in sclerotic skin and diseased lungs of patients suffering from idiopathic pulmonary fibrosis. *Conclusion:* RelA-P-Ser536 may be a core fibrogenic regulator of fibroblast phenotype. (Hepatology 2013)

Liver fibrosis is characterized by excessive deposition of collagenous scar tissue after persistent liver damage. The primary liver-scar–forming cell is the hepatic myofibroblast (HM), which is derived from quiescent hepatic stellate cells (qHSCs).^1^ Liver injury instructs qHSCs to undergo a transdifferentiation program from retinoid-storing cells into HMs, which produce collagen I and enzymes, which prevent collagen degradation, the tissue inhibitors of matrix metalloproteinase.^1^ Continued damage causes perpetuation of the HM phenotype and an imbalance in the deposition and breakdown of fibrotic matrix and, ultimately, the progression of liver fibrosis. Fibrosis in both rodents and humans is a dynamic process that can reverse, as well as progress,^2^ and removal of the injury stimulus or promoting HM apoptosis is associated with fibrolysis.^3^,^4^

Nuclear factor kappa light-chain enhancer of activated B cells (NF-κB) is a transcription factor that contains five subunits, RelA (p65), p50, c-Rel, RelB, and p52, which form either homo-or heterodimers to bind DNA. NF-κB is a master regulator of essential cellular functions, including cell cycle, survival, and immunity.^5^,^6^ The pathway is activated by canonical (RelA, p50, and c-Rel subunits) or noncanonical (RelB and p52 subunit) signaling.^5^

In unstimulated cells, canonical NF-κB is predominantly bound to its inhibitor (IκBα) and retained in the cytoplasm. Stimulation by an appropriate signal, for example, tumor necrosis factor alpha (TNF-α) or lipopolysaccharide (LPS), promotes IκB kinase (IKK)-dependent degradation of IκBα, nuclear translocation of NF-κB, and subsequent DNA binding. In the noncanonical pathway, RelB is bound to p100, the precursor of p52. Stimulation by CD40 or lymphotoxin activates NF-κB-inducing kinase, which then activates IKKα. This promotes the phosphorylation and polyubiquitination of p100, which is then degraded by the proteasome, releasing active p52/RelB.

Canonical NF-κB signaling influences liver fibrosis and promotes HM survival. In models of liver damage, mice lacking *nfkb1* (p105/p50) develop severe inflammation and fibrosis,^7^ whereas *crel* knockout mice develop less fibrosis, but have impaired liver regeneration, compared to wild-type controls.^8^ Pharmacological blockade of NF-κB in HM promotes their apoptosis and enhanced reversal of liver fibrosis.^9^,^10^ However, long-term global NF-κB blockade may alter immune responses or cause cancer.^11^,^12^

In HM, NF-κB is constitutively active. Deregulation of healthy NF-κB activity is controlled by at least two reprogramming events that occur during HSC transdifferentiation. The first is persistent down-regulation in IκBα levels, which is mediated by epigenetic changes,^13^ and the second is phosphorylation of RelA at serine 536 (RelA-P-Ser536).^9^ This post-translational modification promotes RelA nuclear translocation and increases the ability of RelA-containing dimers to interact with coactivators as well as the transcriptional machinery to enhance the transactivation potential of RelA in multiple cells.^14^–^16^ RelA-P-Ser536 is a feature of HM in culture and diseased human liver and can be controlled by autocrine renin-angiotensin system (RAS) signaling.^9^ However, angiotensin blockade does not completely inhibit RelA-P-Ser536 in cultured HM, suggesting that other stimuli or signaling pathways regulate RelA-P-Ser536. Here, we report that in human HM, as with other cells, this modification can be induced by TNF-α stimulation.^15^,^17^ We show that a cell-permeable RelA-P-Ser536 competing peptide (P6) inhibits RelA-P-Ser536 in HM, both *in vitro* and *in vivo*, and has antifibrotic, but not anti-inflammatory, effects in multiple models of liver injury.

## Materials and Methods

Reagents are listed in the Supporting Materials.

### Cell Culture

Human HMs were isolated from livers of adult male patients after surgical resection under Ethics Committee approval, subject to patient consent.^9^ LX2 cells were from Prof. Dr. Scott Friedman (Mount Sinai School of Medicine, New York, NY). Nonalcoholic steatohepatitis (NASH), idiopathic pulmonary fibrosis (IPF), and scleroderma samples were taken under full ethical approval and patient consent (REC references 10/H0906/41, 11/NE/0291 and 09/H0905/11). Human lung epithelial cells were plated in collagen-coated dishes and cultured with Small Airway Epithelial Cell Basal Medium (SABM™; Lonza, Basel, Switzerland). HM and LX2 were cultured in Dulbecco’s modified Eagle’s medium, human lung fibroblasts were cultured with modified Eagle’s medium, and human skin fibroblasts were cultured in Iscove’s modified Eagles medium, supplemented with 100 U/mL of penicillin, 100 µg/mL of streptomycin, 2 mM of L-glutamine, and 16% fetal bovine serum (FBS) and were maintained at 37°C in an atmosphere of 5% CO_2_.

### In Vivo *Models of Rodent Liver Fibrosis and Sublethal* LPS

Mice (8-10 weeks old; C57BL/6) were purchased from Harlan Laboratories (Indianapolis, Indiana). Acute CCl_4_ with cell-permeable peptides was produced as previously described.^9^ Chronic CCl_4_ was injected intraperitoneally (IP) biweekly at 2 μL (CCl_4_/olive oil, 1:3 [v/v])/g/body) for 8 weeks. From 3 weeks, mice received DM (control), P6 (competing) peptide (Supporting Table 1) (10 mg/kg), or vehicle triweekly by IP injection for a further 5 weeks. Mice were fed a methionine-choline-deficient (MCD) or control diet for 2 weeks, then received DM, P6 peptide (10 mg/kg), or vehicle triweekly by IP injection and diet for a further 5 weeks. Pure LPS (InvivoGen, San Diego, CA) was administered by IP injection at a dose of 300 µg/animal for 24 hours. Mice were given IKK-2 Inhibitor VI (5 mg/kg; Merck KGaA, Darmstadt, Germany), P6 peptide (10 mg/kg), or vehicle by IP injection 1 hour before and 6 hours after LPS administration.

### Immunohistochemistry

Immunohistochemistry was performed on formalin-fixed liver sections. Alpha smooth muscle actin (α-SMA), CD3, and anti-neutrophil antibody (NIMP-R14) were performed as previously described.^8^ For F4/80 (Abcam, Cambridge, MA) or RelA-P-Ser536 (Cell Signaling Technologies, Inc., Danvers, MA, or Abnova, Taipei City, Taiwan) and cleaved caspase-3 (Cell Signaling Technologies), deparaffinized sections were incubated in hydrogen peroxide/methanol. Proteinase K (20 µg/mL) or citrate saline antigen retrieval were performed. Endogenous avidin and biotin were blocked using the Vector Avidin/Biotin Blocking Kit (Vector Laboratories, Inc., Burlingame, CA), and further blocking was achieved using swine serum. Sections were incubated overnight with primary antibodies (Abs). On the next day, sections were washed and incubated with biotinylated swine antirabbit (1:200), followed by VECTASTAIN Elite ABC Reagent (Vector Laboratories). Antigens were visualized using diaminobenzidine peroxidase substrate and counterstained with Mayer’s hematoxylin.

### Hematoxylin and Eosin, Sirius Red, and Oil Red O Staining

Formalin-fixed liver sections were stained with hematoxylin and eosin (H&E) and 0.1% Sirius Red Picric solution, following standard procedures. Isopentane-preserved liver sections were fixed with Baker’s formol calcium, stained with 60 mg/mL of Oil Red O in isopropanol, and mounted with aqueous mounting medium.

### Cell Counts and Image Analysis

Slides were blinded, and at least 15 fields were manually counted for NIMP-1 and CD3 staining at ×20 magnification. Image analysis of 10 fields was performed using Leica QWin software (Leica Microsystems Inc., Buffalo, IL) for α-SMA, Sirius Red, Oil Red O, and F4/80 staining at ×10, unless otherwise stated.

### Immunoprecipitation

Cells were lysed with 20 mM of Hepes, 400 mM of NaCl, 1 mM of ethylenediaminetetraacetic acid, 25% glycerol, and 0.1% NP-40 buffer. Lysates were precleared for 2 hours, then incubated overnight with 5 µg of Ab or immunoglobulin G). Recombinant Protein G beads were added to samples and incubated for 1.5 hours. Beads were washed and eluted with Laemmli buffer.

### Kinase Assay

Glutathione *S*-transferase (GST) and GST/RelA recombinant fusion proteins were from Prof. Neil Perkins (Newcastle University, Newcastle upon Tyne, UK). GST and GST/RelA fusion proteins were incubated with glutathione agarose beads for 2 hours at 4°C. Beads were pulled down and incubated in 50 mM of Tris buffer (pH 7.4), 100 mM of NaCl, 5 mM of MgCl_2_, and 1 mM of dithiothreirol with 0.5 mM of adenosine triphosphate, 1.5 µL of recombinant IKKα/β, and 10 µL of peptides (10 mM) at 30°C for 40 minutes. Kinase assay supernatant was collected for western blotting analysis. Beads were rinsed and eluted with Laemmli buffer.

### RNA Isolation and Real-Time Polymerase Chain Reaction

Total RNA was isolated from HM or liver tissue with TRI Reagent. The aqueous phase was extracted and RNA was precipitated with isopropanol, then washed with 70% alcohol before quantification by NanoDrop. Complementary DNA synthesis was performed using a Promega kit (Promega Corporation, Madison, WI). Real-time polymerase chain reaction was performed with SYBR Green JumpStart Taq ready mix, following the manufacturer’s instructions. Primer sequences are shown in Supporting Tables 2 and 3.

### Statistical Analysis

*P* values were calculated using a two-tailed paired Student *t* test or a one-way analysis of variance, and *P* < 0.05 or *P* < 0.01 was considered significant.

## Results

### The RelA Phosphorylation Competing Peptide P6 Is an IKK Substrate, Not IKK Inhibitor, *In Vitro*

**Fig 1:**
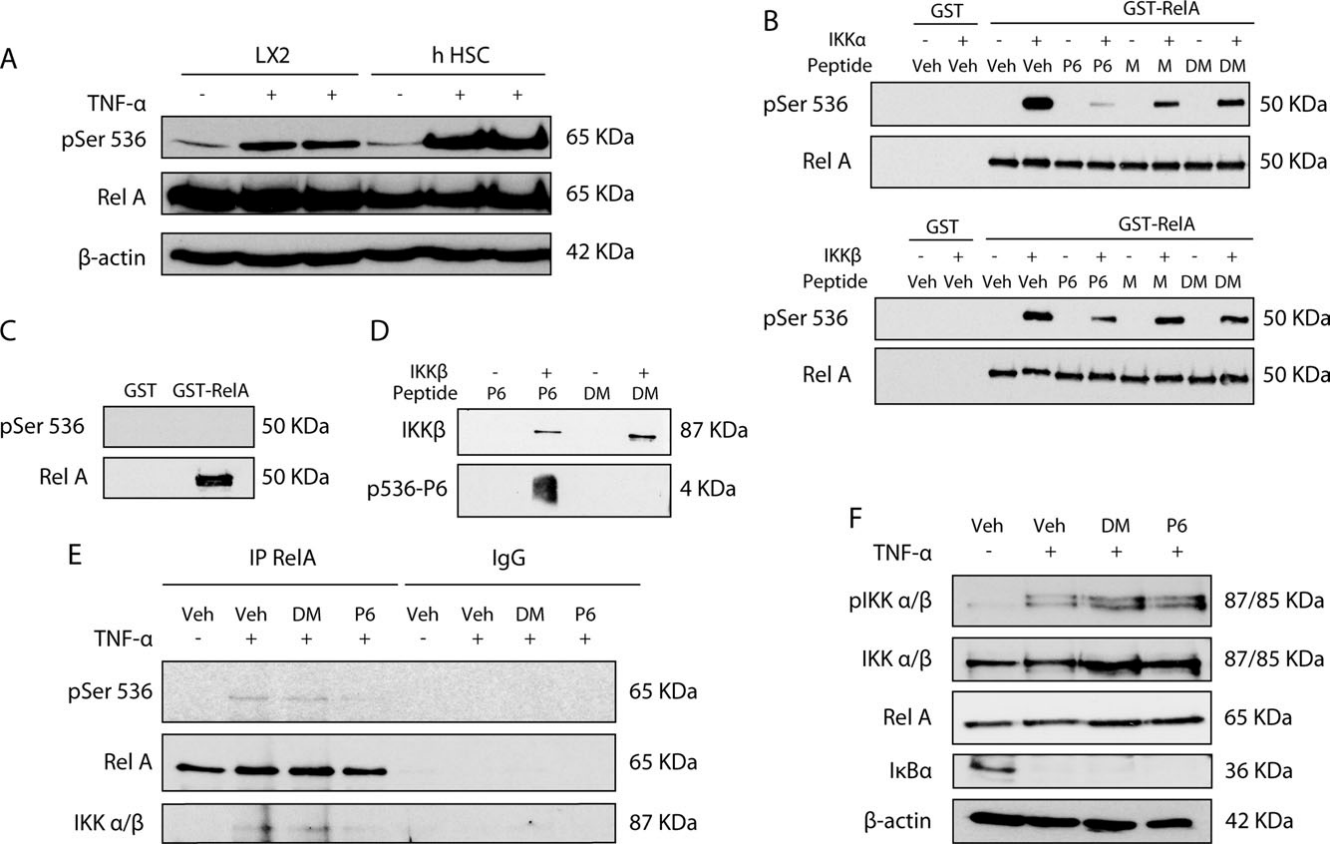
P6 is a competing peptide, not an IKK inhibitor. Western blotting showing increased RelA-P-Ser536 in LX2 and hHM cultured in 0.5% FBS after stimulation with 100 ng/mL of TNF-α (A). P6 reduces GST-RelA-P-Ser536 by IKK-α and-β (B) in an *in vitro* kinase assay. Kinase assay input control (C). Western blotting of IKK-β kinase assay supernatant shows that P6 is phosphorylated at Ser536 by IKK-β (D). Iimmunoprecipitation for RelA in passage 4 hHM ± 100 µM of peptides for 1 hour, then stimulated with 100 ng/mL of TNF-α for 10 minutes (E). Input control for RelA immunoprecipitation (F). Gels are representative of at least three separate experiments.

HM RelA-P-Ser536 is regulated by the RAS; however, blocking this pathway does not completely inhibit this phosphorylation event, so other regulatory mechanisms must exist. HMs, in injured livers, are exposed to multiple cytokines and inflammatory mediators, including TNF-α. RelA-P-Ser536 is induced by TNF-α in multiple cell types,^17^–^20^ so we asked whether TNF-α promotes RelA-P-Ser536 in HMs. TNF-α stimulation of serum-starved LX2 cells and human HM (hHM) rapidly induced RelA-P-Ser536 (Fig. 1A), highlighting this as an additional regulatory pathway. We previously used a cell-permeable peptide (P6) spanning amino acids 525-536 of RelA linked to a penetratin domain to selectively target RelA-P-Ser536. The peptide is reported to compete with endogenous RelA for phosphorylation^21^; however, its mechanism of action in HM has not been fully investigated. P6 could have off-target effects and could function as an IKK inhibitor, blunting all NF-κB signaling, including innate immune response. This would be undesirable, because NF-κB pan-blockade could promote hepatocyte death and liver cancer and offer no advantage over current IKK inhibitors.^22^–^24^ Therefore, we performed *in vitro* kinase assays to test whether P6 inhibits IKK activity. P6 reduces the phosphorylation of GST/RelA at Ser536 by either recombinant IKKα/β, compared to the control peptides, M (contains Ser529, but Ser536 is substituted to alanine) or DM (Ser529 and Ser536 are replaced with nonphosphorylatable alanine) (Fig. 1B). DM was used as a control peptide because Ser529 phosphorylation may act synergistically with Ser536 to recruit kinases or other interacting proteins. The addition of M and DM reduced IKKα-dependant GST/RelA-P at Ser536, compared to vehicle (no peptide), suggesting some competition or steric hindrance of IKKα by peptide; however, GST/RelA-Ser536-P levels were further reduced upon P6 addition. GST/RelA is not phosphorylated at Ser536 in the absence of the kinase (Fig. 1C). Western blotting for RelA-P-Ser536 revealed that P6, not DM, was a substrate for IKKβ *in vitro*, confirming the peptide targeted by IKK for phosphorylation, and is not an IKK inhibitor (Fig. 1D). To recapitulate this *in vitro*, we pretreated hHM with peptide, then stimulated this with TNF-α and performed an immunoprecipitation for RelA. P6, but not DM, prevented RelA-P-Ser536 (Fig. 1E). We could coimmunoprecipitate IKKα/β with RelA in the vehicle and DM groups, but this interaction was reduced upon P6 treatment, suggesting that the peptide disrupts RelA/IKK complex formation. Whole-cell input controls from the RelA immunoprecipitation proved that, in the presence of P6 or DM, TNF-α promotes healthy IKK activation, as shown by phosphorylated IKKα/β and degradation of the IKK substrate, IκBα (Fig. 1F), confirming that P6 is targeted by IKK for phosphorylation and does not impair IKK activation.

### P6 Does Not Impair Innate Immune Response After LPS Challenge

**Fig 2:**
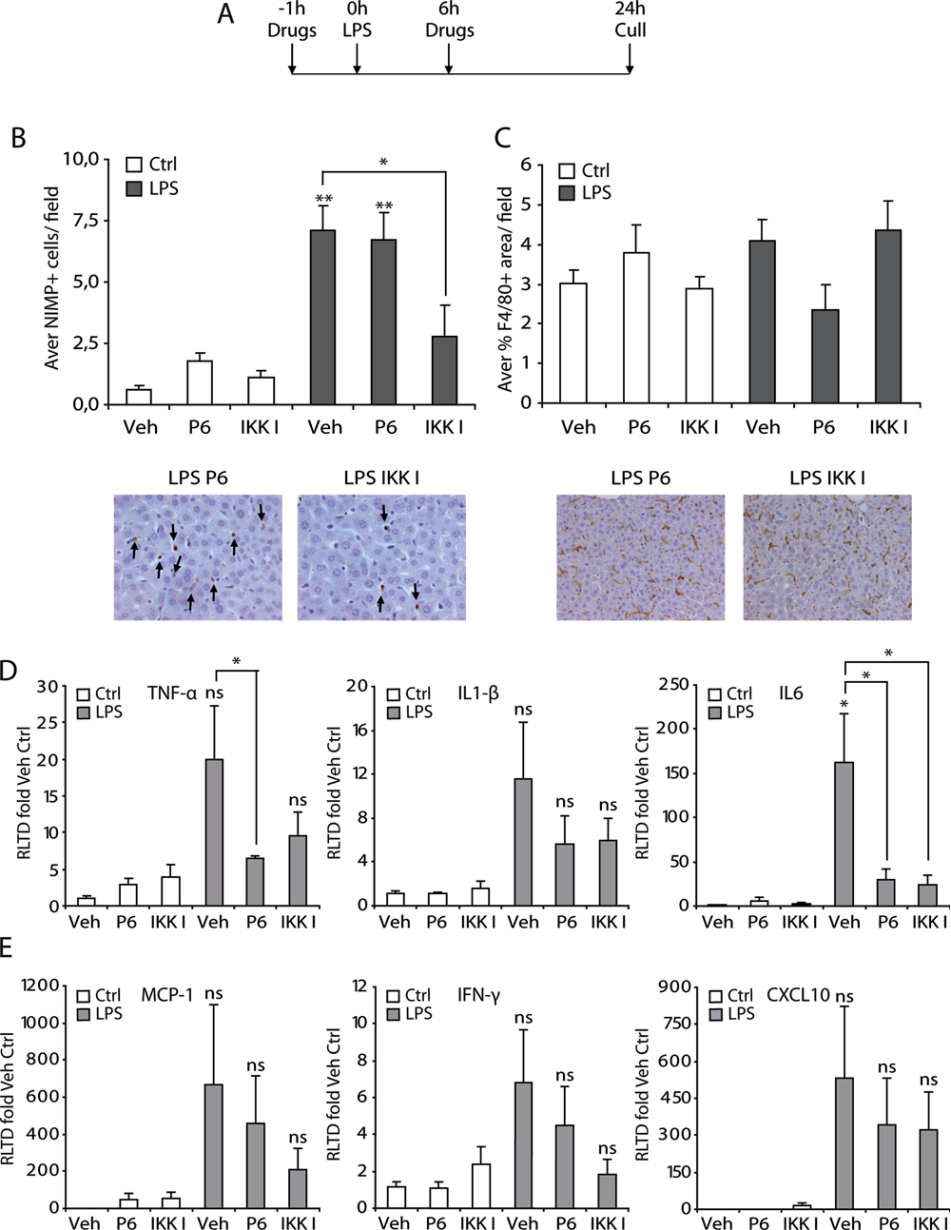
IKK inhibitor, but not P6, blocks innate immune response in a systemic sublethal LPS sepsis model. Experimental model design (A). Quantification of hepatic neutrophil (NIMP) (B) and macrophage (F4/80) (C), representative photomicrographs at ×400 and ×200 magnification, respectively. Hepatic TNF-α, IL1-β, IL6 (D), MCP-1, IFN-γ, and CXCL10 (E) mRNA expression. Data are expressed as mean ± standard error of the mean and are representative of 5 animals per group.

NF-κB regulates inflammation; therefore, globally blocking this pathway could have severe adverse effects and could impair innate immune responses. Previous studies that inhibited IKK signaling using either pharmacological agents or genetically modified mice have revealed aberrant (i.e., neutrophilia) or weakened inflammatory responses.^11^,^23^ LPS promotes IKKβ-dependent RelA-P-Ser536 in multiple cells.^25^,^26^ To determine whether targeting RelA-P-Ser536 using P6 compromises this response, we challenged mice with a sublethal LPS dose and treated them with either P6 or IKK inhibitor (Fig. 2A).

The IKK inhibitor reduced hepatic neutrophil infiltration at 24 hours after LPS administration; conversely, P6 had no effect (Fig. 2B). Hepatic F4/80^+^ macrophages and CD3^+^ T-cell numbers were unchanged upon administration of LPS or therapy (Fig. 2C and Supporting Fig. 1A). Cytokine and chemokine induction are central to the initiation of an innate immune response and hepatic neutrophil recruitment. P6 and IKK inhibitor reduced liver TNF-α, interleukin (IL)-1β, IL-6, macrophage inflammatory protein (MIP)-2α, and regulated upon activation, normal T-cell expressed, and secreted (RANTES) expression (Fig. 2D and Supporting Fig. 1B). Conversely, the induction of monocyte chemotactic protein 1 (MCP-1) and the critical driver of innate immunity interferon-gamma (IFN-γ) was suppressed by the IKK inhibitor, but unaffected by P6, whereas CXC chemokine ligand 10 (CXCL10) (IP-10) was unchanged (Fig. 2E). This suggests that P6 and IKK inhibitors differentially regulate NF-κB-dependent genes.

### P6 Peptide Does Not Affect Hepatic Inflammation in Response to Acute Liver Injury

**Fig 3:**
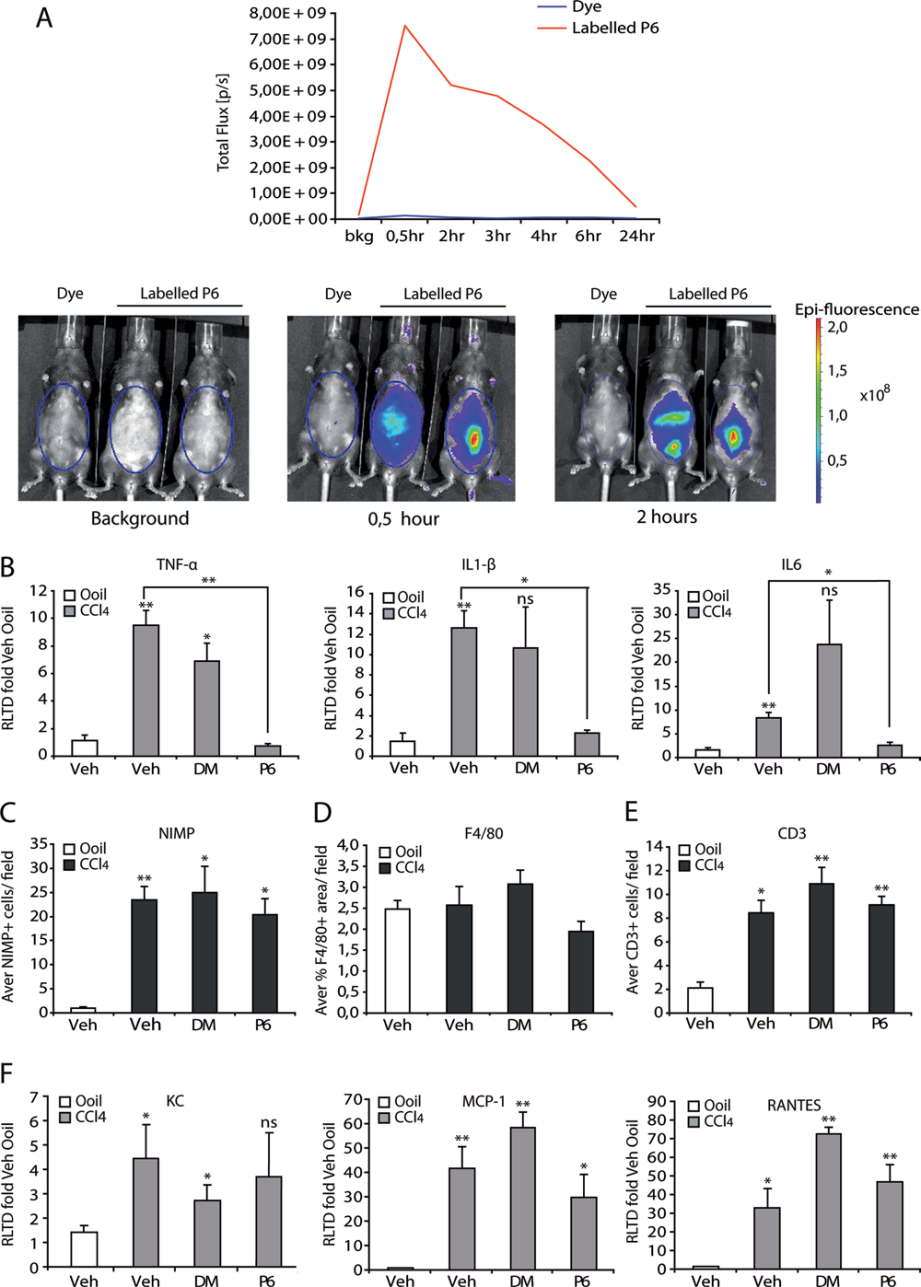
P6 does not suppress inflammation in acute CCl_4_ injury. IVIS tracking of DyLight 800 fluorescent-labeled P6 up to 24 hours IP injection. Graph shows total flux [p/s] taken from the regions of interest at 0.5, 2, 3, 4, 6, and 24 hours; n = 2 mice. Free dye was used as a control. Representative images of background 30 minutes and 2 hours after immunoprecipitation. Blue ovals denote the regions of interest used to calculate total flux. (A) Hepatic TNF-α, IL-1β, and IL-6 mRNA expression (B). Quantification of neutrophils (NIMP), (C) macrophages (F4/80) (D), and hepatic T cells (CD3) (E). Whole-liver mRNA expression of KC, MCP-1, and RANTES (F). Significance is compared to vehicle, unless shown on graph. Data are expressed as mean ± standard error of the mean and are representative of 5 animals per group.

We reported that P6 reduced fibrogenic gene expression in acute CCl_4_ toxic liver injury.^9^ Given our observations in the LPS model, we decided to investigate the effect of P6 on inflammation in this model. To assess P6 bioavailabilty and distribution after IP injection, we used *in vitro* injection system (IVIS) imaging to track fluorescent-labeled P6 (Fig. 3A). Thirty minutes after administration, fluorescent P6 was detected throughout the abdominal cavity, but within 2 hours, the signal localized to the upper abdomen and liver. The signal was reduced by ∼50% over the next 4 hours and almost gone by 24 hours. *Ex vivo* imaging of internal organs showed retention of peptide within the liver and kidneys; however, no signal could be detected in the spleen (Supporting Fig. 2A, B). Serum transaminase alanine aminotransferase (ALT) was elevated equally in all groups, confirming that P6 did not affect liver injury (Supporting Fig. 2C). P6, but not DM, reduced TNF-α, IL-1β, IL-6, and MIP-2α expression (Fig. 3B and Supporting Fig. 2D); however, hepatic neutrophil, macrophage, and T-cell numbers were unchanged (Fig. 3C-E). Then, we measured hepatic levels of other NF-κB-regulated inflammatory genes and found no difference in KC, MCP-1, and RANTES expression (Fig. 3F). These chemokines may compensate for the loss of TNF-α, IL-1β, IL-6, and MIP-2α, providing further evidence that RelA-P-Ser536 signaling only regulates a subset of NF-κB-dependent genes.

### RelA-P-Ser536 Is Not Required for RelA Nuclear Translocation in hHM

**Fig 4:**
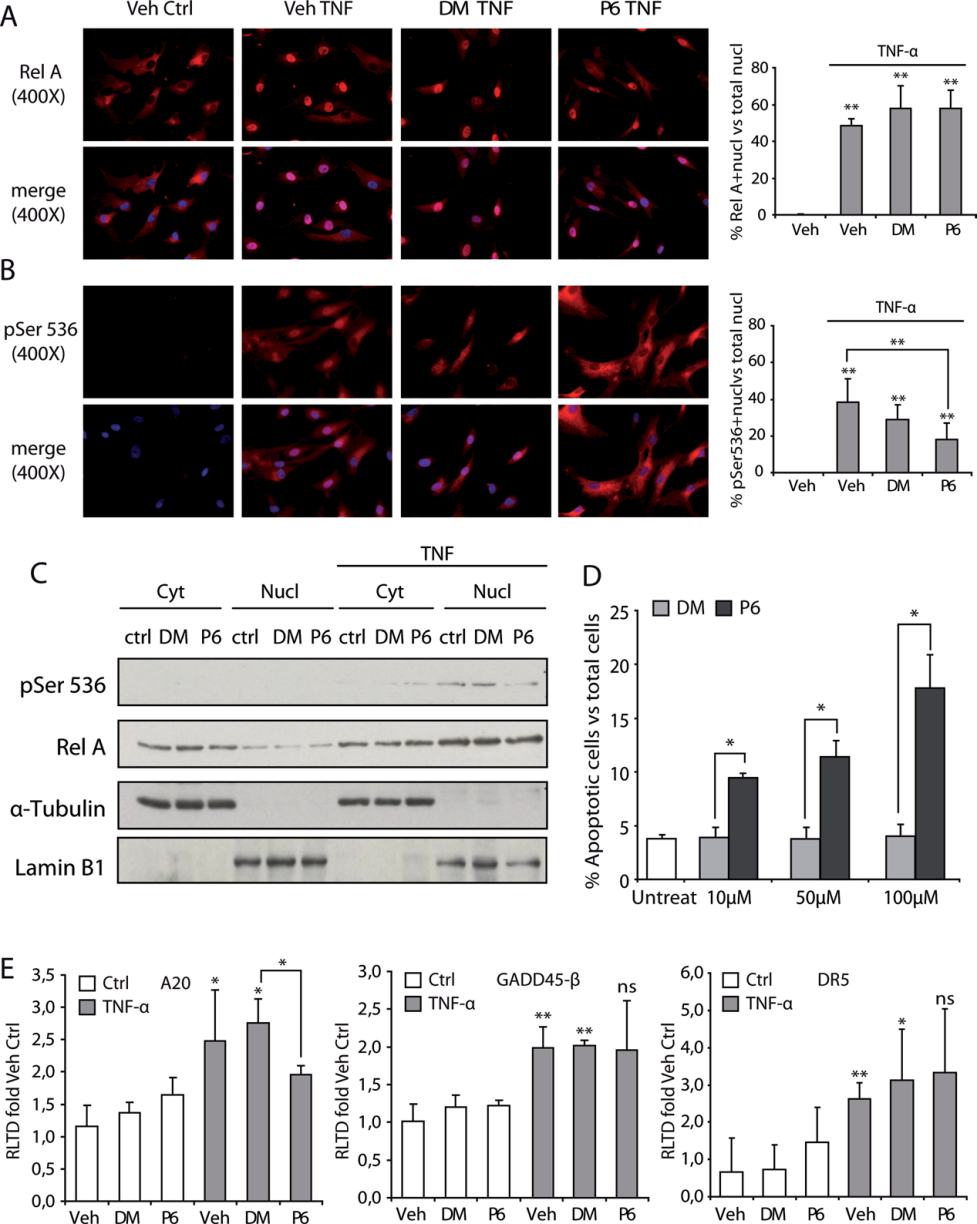
RelA nuclear translocation is RelA-P-Ser536 independent in hepatic myofibroblasts. Epifluorescence images at ×400 magnification and quantification of nuclear translocation in hHMs of RelA (red) (A) or RelA-P-Ser536 (red) (B) and 4′,6-diamidino-phenylindole (blue) in P4 hHMs in 0.5% serum-stimulated ± 100 µM of peptides for 1 hour and 50 ng/mL of TNF-α for 10 minutes. Western blotting of cytoplasmic (Cyt) and nuclear (Nucl) extracts isolated from LX2-cells–stimulated ± 100 µM of peptides and TNF-α (C). Acridine orange apoptosis assay in hHMs ± 10-100 µM of peptides 24 hours (D). A20, GADD45-β, and DR5 gene expression in LX2 cells in 0.5% serum-stimulated ± 100 µM of peptides overnight and 50 ng/mL of TNF-α for 5 hours (E). Data are expressed as mean ± standard deviation, compared to control group, and is representative of at least three separate experiments.

RelA-P-Ser536 is required for effective nuclear translocation of RelA upon stimulation with serum in serum-starved vascular smooth muscle cells,^27^ and RelA/enhanced green fluorescent protein reconstituted RelA-null mouse embryonic fibroblasts.^9^ However, this was not the case in TNF-α-stimulated hHM under serum-free conditions. TNF-α-induced RelA nuclear translocation in hHM was not blocked by DM or P6 (Fig. 4A). However, RelA-P-Ser536 was reduced in the nuclei of P6-treated cells, compared to vehicle or DM (Fig. 4B). RelA-P-Ser536 levels in nuclear and cytoplasmic extracts isolated from LX2 cells treated with or without TNF-α and DM or P6 confirmed this (Fig. 4C). In hHM, P6 inhibits RelA-P-Ser536, but not RelA, nuclear translocation, which provides an explanation as to why P6 only affects a subset, and not all, of NF-κB-regulated inflammatory genes. Akin to rat HMs, hHMs undergo a dose-dependent apoptosis when treated with P6, but not DM (Fig. 4D). TNF-α activates NF-κB and, subsequently, antiapoptotic gene expression, but initiates proapoptotic signaling cascades in parallel (Jun N-terminal kinase and caspase-8 activation); therefore, cell fate is determined by subtle imbalances in pro-and antiapoptotic genes.^28^ A20, a gene target of RelA-P-Ser536, has dual functions, in that it prevents NF-κB activation, but conveys protection against TNF-α-dependent apoptosis.^29^ P6 reduces A20 expression in TNF-α-stimulated LX2, providing one explanation for the proapoptotic effects of the peptide; interestingly, antiapoptotic GADD45β and proapoptotic DR5, BAX, and NOXA were unchanged (Fig. 4E and Supporting 3A, B). P6 does not affect α-SMA or Col1A1 expression in TNF-α-stimulated LX2 (Supporting 3C, D).

### Blocking RelA-P-Ser536 Is Antifibrotic, but Not Anti-inflammatory, in Progressive Liver Injury

**Fig 5:**
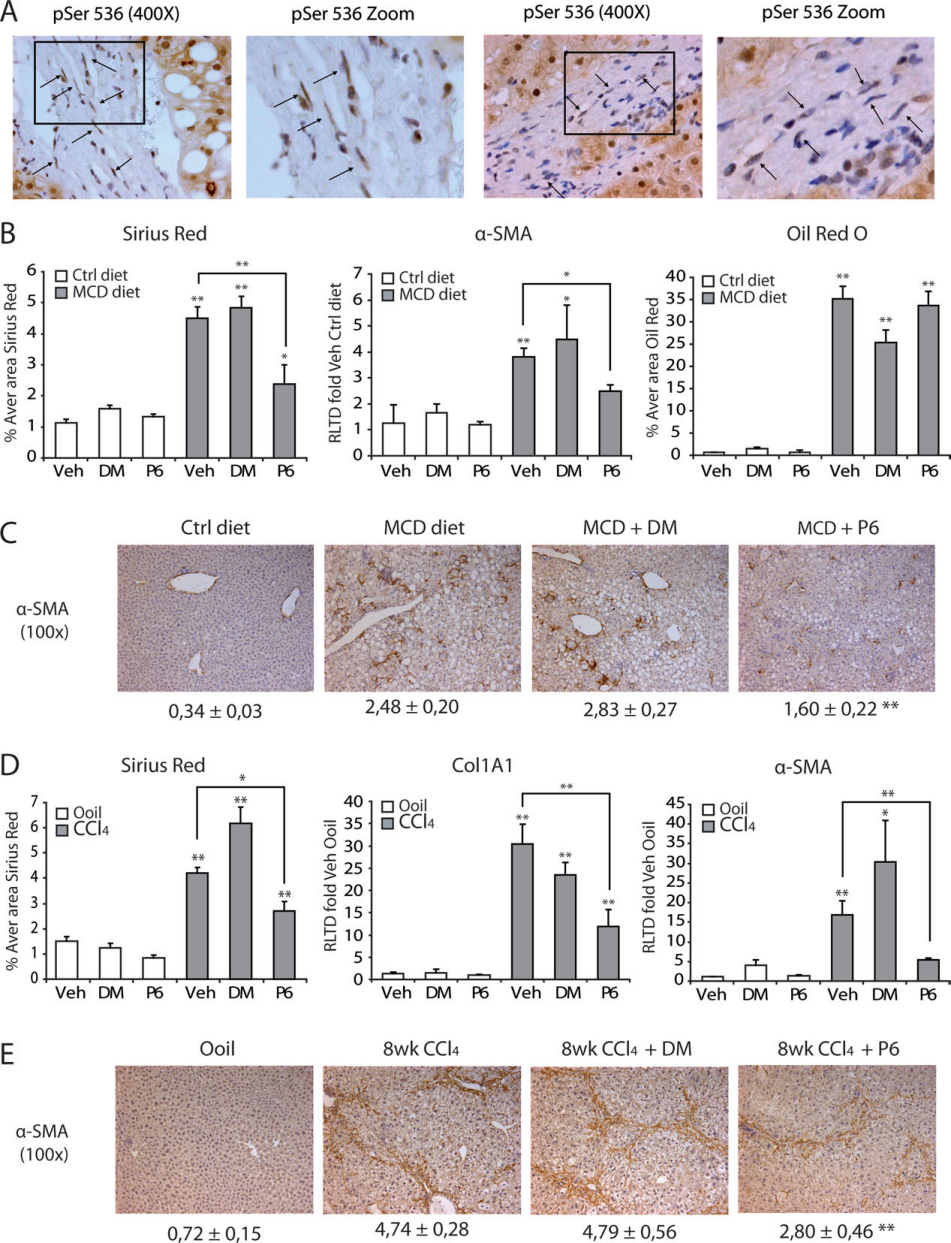
P6 is antifibrotic in two models of progressive liver injury. RelA-P-Ser536 staining in fibrotic bands in NASH patients (A); black arrows denote RelA-P-Ser536-stained cells. Quantification of liver Sirius Red staining, α-SMA mRNA expression, and hepatic Oil Red O staining (B) in the MCD diet model. Representative photomicrographs of α-SMA-stained (C) liver sections in the MCD diet model. Quantification of Sirius Red staining in the chronic CCl_4_ injury model, hepatic collagen and α-SMA mRNA expression (D), and α-SMA staining in liver sections (E) in the chronic CCl_4_ model. Data are expressed as mean ± standard error of the mean of five animals per group.

Antifibrotic therapies need to be effective in situations of ongoing liver injury once disease is established. To determine whether inhibiting RelA-P-Ser536 signaling using P6 is antifibrotic under these conditions, we tested the peptide in the MCD and CCl_4_ liver injury models. MCD is histologically similar to NASH, so we stained NASH patients for RelA-P-Ser536. HMs in NASH livers are RelA-P-Ser536 positive; however, we also observed strong immunoreactivity in some hepatocytes and biliary cells (Fig. 5A). Mice were fed an MCD diet for 2 weeks to induce steatohepatitis with mild fibrosis, then given peptides triweekly for 5 weeks alongside MCD. Similar to NASH patients, RelA-P-Ser536-positive HMs were detected in livers of MCD-fed animals. Although some hepatocyte nuclei were RelA-P-Ser536 positive, immunoreactivity in the mouse was much lower than in humans (Supporting Fig. 4A). P6 therapy significantly reduced fibrosis, demonstrated by a reduction in collagen deposition (Sirius Red), α-SMA messenger RNA (mRNA), and α-SMA^+^ cells (Fig. 5B-C). However, no changes in hepatic Col1A1 mRNA levels were observed (Supporting Fig. 4B). Fat deposition (Oil Red O staining) (Fig. 5B), liver damage assessed by serum ALT, H&E, and active caspase-3 staining (Supporting Fig. 5A-C) were similar across all treatment groups, confirming that P6 did not affect the injury process. Next, we tested P6 in a second chronic liver disease model. We gave CCl_4_ for 3 weeks to induce mild fibrosis, then coadministered the DM and P6 peptides for a further 5 weeks. Treatment with either peptide did not affect ALT levels, histological liver injury, or hepatocyte apoptosis (H&E and active caspase-3) (Supporting 5D-F). Dual immunofluorescence staining for α-SMA and RelA-P-Ser536 revealed colocalization of both antigens in 8-week CCl_4_-injured livers. RelA-P-Ser536 immunoreactivity was lost upon P6 treatment, and the remaining α-SMA^+^ cells were RelA-P-Ser536 negative (Supporting Fig. 6A), confirming that P6 inhibits HM RelA-P-Ser536 *in vivo*. Sirius Red morphometry and hepatic collagen gene expression confirmed a reduction in collagen deposition in P6-treated animals (Fig. 5D). This was accompanied by decreased hepatic α-SMA gene expression and staining (Fig. 5D-E). Next, we asked whether P6 affected hepatic inflammation in either model. We observed no difference in T-cell or neutrophil recruitment to the MCD-injured liver after P6 therapy (Supporting Fig. 6B-C). TNF-α and IL-1β were slightly reduced, but RANTES and MCP-1 expression remained unchanged (Supporting Fig. 6D). P6 did not affect neutrophil recruitment, but did reduce the number of CD3^+^ T cells in the CCl_4_ model (Supporting 6E-F). P6 induces HM apoptosis *in vitro*; therefore, the antifibrotic effects could be the result of HM apoptosis, rather than suppressing inflammation.

### RelA-P-Ser536 Is a Feature of Lung Fibroblasts and IPF

**Fig 6:**
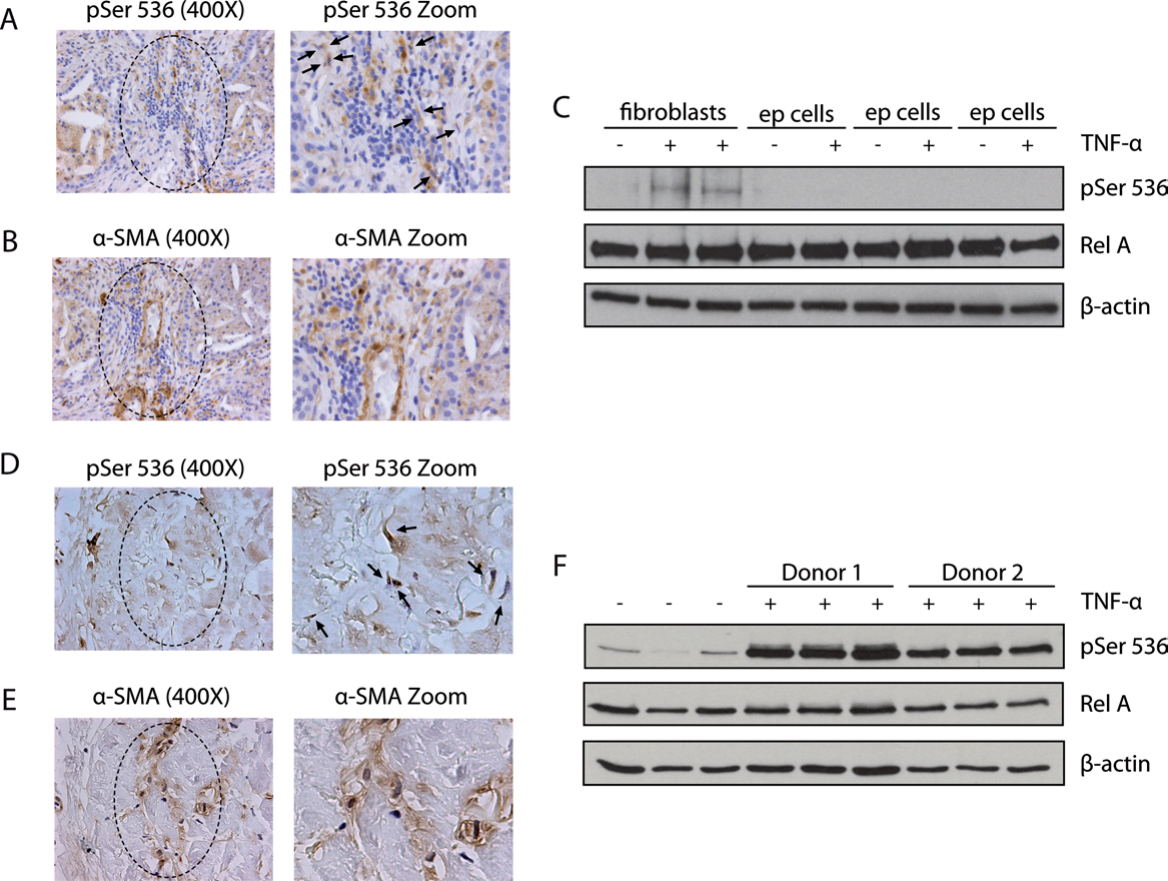
RelA-P-Ser536 is a feature of human lung and skin fibroblasts and is regulated by TNF-α. Representative photomicrographs of RelA-P-Ser536 (A) and α-SMA (B) staining in human IPF slides; black arrows denote RelA-P-Ser536-stained cells. Western blotting showing RelA-P-Ser536 and total RelA in passage 3 human lung fibroblasts or epithelial cells cultured with 0.5% FBS and stimulated with 50 ng/mL of TNF-α for 10 minutes (C). Representative photomicrographs of RelA-P-Ser536 (D) and α-SMA (E) staining of human consecutive slides of sclerodema; black arrows denote RelA-P-Ser536-stained cells. Western blotting showing RelA-P-Ser536 and total RelA human healthy skin fibroblasts cultured with 0.5% FBS and stimulated with 50 ng/mL of TNF-α for 10 minutes (F). Data represent at least two separate cell lines and 3 IPF and scleroderma patients.

A recent review highlighted the importance of identifying “core” fibrogenic pathways in multiple organs.^30^ To determine whether commonality exists in the signaling pathways of fibroblasts in liver and lung fibrosis, we performed RelA-P-Ser536 staining in IPF lung patients. We observed RelA-P-Ser536 positivity in fibrotic areas of IPF lungs, which follows a similar distribution to α-SMA^+^ staining (Fig. 6A,B). Next, we next performed western blotting analysis for RelA-P-Ser536 in human lung fibroblasts and epithelial cells. Akin to hHM and LX2, stimulation of serum-starved human lung fibroblasts with TNF-α increased RelA-P-Ser536; conversely, there was little or no induction in epithelial cells (Fig. 6C and Supporting Fig. 7). In addition to our observations, Reber et al. reported that RelA is phosphorylated at Ser276 and Ser536 in IL-1β-stimulated lung fibroblasts.^31^ We could also induce RelA-P-Ser536 by TNF-α in healthy human skin fibroblasts and observe this modification in dermal fibroblasts in patients with scleroderma (Fig. 6D-F). Multiple stimuli promote RelA-P-Ser536 in human liver, lung, and skin fibroblasts, suggesting a degree of commonality in fibrotic disease in these organs.

## Discussion

NF-κB signaling regulates wound healing and inflammation^5^,^22^; therefore, long-term pan-blockade in the liver could have detrimental side effects. However, the biological outcomes of NF-κB activation are dependent on numerous factors, and regulatory checkpoints exist at each step of the signaling pathway, including stimuli, activating kinase, dimer composition, post-translational modifications, and DNA-binding consensus sequence.^5^,^14^ We focused on a single post-translational modification and provide evidence that RelA-P-Ser536-dependent signaling in HM causes fibrosis during chronic liver injury, but is not a critical regulator of hepatic inflammation during liver injury or LPS challenge. However, targeting RelA phosphorylation generally or focusing on additional sites could have therapeutic potential. This is consistent with the idea that multiple post-translational modifications of NF-κB subunits, including RelA, exist to subtly modulate NF-κB signaling in response to different stimuli. RelA phosphorylation at Ser276, Ser311, Ser468, Ser529, and Ser536 are “activating” modifications,^15^,^31^–^34^ whereas Thr505 is “repressive”^35^; however, Ser468 and Ser536 can be either repressive or activating, depending on stimuli and cell type.^26^,^36^ For instance, in macrophages, LPS-induced RelA-P-Ser536 by IKKα causes an accelerated turnover of RelA on target genes and attenuates the inflammatory response.^26^ Conversely, in neonatal cardiomyocytes, activation of IKKβ by LPS induces RelA-P-Ser536 and promotes its recruitment to the TNF-α promoter, resulting in an elevated, prolonged induction of TNF-α gene expression.^37^ In HMs, P6 reduced RelA-P-Ser536, but did not prevent RelA nuclear translocation, and P6 administration *in vivo* regulates only a subset of inflammatory genes. In LPS challenge and liver fibrosis models, the proinflammatory cytokines MCP-1, KC, and RANTES were normally induced and able to compensate for loss of TNF-α and IL-6 and elicit a normal inflammatory response. Similarly to our results, phosphorylation of Ser276^32^ or Ser536^19^ sites in RelA can also differentially regulate the transcription of NF-κB-dependent subset of genes in HeLa or Jurkat cells. Importantly, we demonstrated that in hHM, P6 competes with RelA for phosphorylation, is not an IKK inhibitor, and does not impair normal NF-κB activation. We cannot rule out the possibility that P6 disrupts RelA complexes with IKK, other kinases or coactivators, or changes dimer composition to exert its effects. For example, other regulatory kinases, including IKKε^20^ and TANK-binding kinase 1 (TBK1), can phosphorylate RelA at Ser536 in response to TNF-α stimulation.^17^,^38^ Buss et al. showed that TBK1-mediated phosphorylation at this site results in the destruction of a hydrogen bond with Asp533, reducing its affinity for the corepressor, amino-terminal enhancer of split, but increasing its affinity for the transcriptional coactivator, TAFII31.^38^ Equally, we cannot exclude the possibility that synergy exists between Ser529 and Ser536, both of which are present in P6 and may be required to recruit or stabilize RelA/kinase interactions. P6 is antifibrotic in two etiologically distinct models of progressive injury, suggesting that this may be a core signaling event in HM. Fibrosis is reported to be the underlying cause of up to 40% of deaths in the western world, and, as such, generic or common targets are a priority for antifibrotic drug development. We report that RelA-P-Ser536 is stimulated by TNF-α in human lung fibroblasts, but not lung epithelial cells, and observed RelA-P-Ser536 immunoreactivity in fibrotic areas of patients with IPF. Similarly, this modification can be induced in healthy skin fibroblasts by TNF-α, and RelA-P-Ser536 myofibroblast-like positive cells are localized in fibrotic areas in scleroderma patients. RelA-P-Ser536 could be a fibrogenic regulator in multiple organs, and further investigation is warranted. Peptides are not a clinically viable therapeutic option resulting from problems associated with the development of autoimmunity. Selectively blocking RelA-P-Ser536 using small-molecule inhibitors or exploiting cell-targeting vehicles, such as liposomes and albumin based carriers,^39^ to deliver peptides to HM may provide potential alternative strategies. Alternatively, focusing on RelA-P-Ser536 target genes may be beneficial. Selectively targeting RelA-P-Ser536 may allow HM clearance and resolution or halting of fibrosis, but retain the ability to mount an inflammatory response.

## Abbreviations

Ab: antibody
α-SMA: alpha smooth muscle actin
ALT: alanine aminotransferase
CXCL10: CXC chemokine ligand 10
FBS: fetal bovine serum
GST: glutathione *S*-transferase
H&E: hematoxylin and eosin
HM: hepatic myofibroblast
hHM: human HM
IFN-γ: interferon-gamma
IKK: IκB kinase
IL: interleukin
IP: intraperitoneal
IPF: idiopathic pulmonary fibrosis
IVIS: *in vivo* imaging system
LPS: lipopolysaccharide
MCD diet: methionine-choline–deficient
MCP-1: monocyte chemotactic protein 1
MIP: macrophage inflammatory protein
mRNA: messenger RNA
NASH: nonalcoholic steatohepatitis
NF-κB: nuclear factor kappa light-chain enhancer of activated B cells
NIMP-1: Nogo-interacting mitochondrial protein 1
qHSCs: quiescent hepatic stellate cells
RANTES: regulated upon activation, normal T-cell expressed, and secreted
RAS: renin-angiotensin system
RelA-P-Ser536: phosphorylation of RelA subunit at serine 536
TBK1: TANK-binding kinase 1
TNF-α: tumor necrosis factor alpha.
