# Supplementary figures and images for: Inhibition of RelA-Ser536 Phosphorylation by a Competing Peptide Reduces Mouse Liver Fibrosis Without Blocking the Innate Immune Response
Source: Hepatology. 2013 Jan 8;57(2):817–28. doi: 10.1002/hep.26068 (PMC3807604; doi:10.1002/hep.26068)

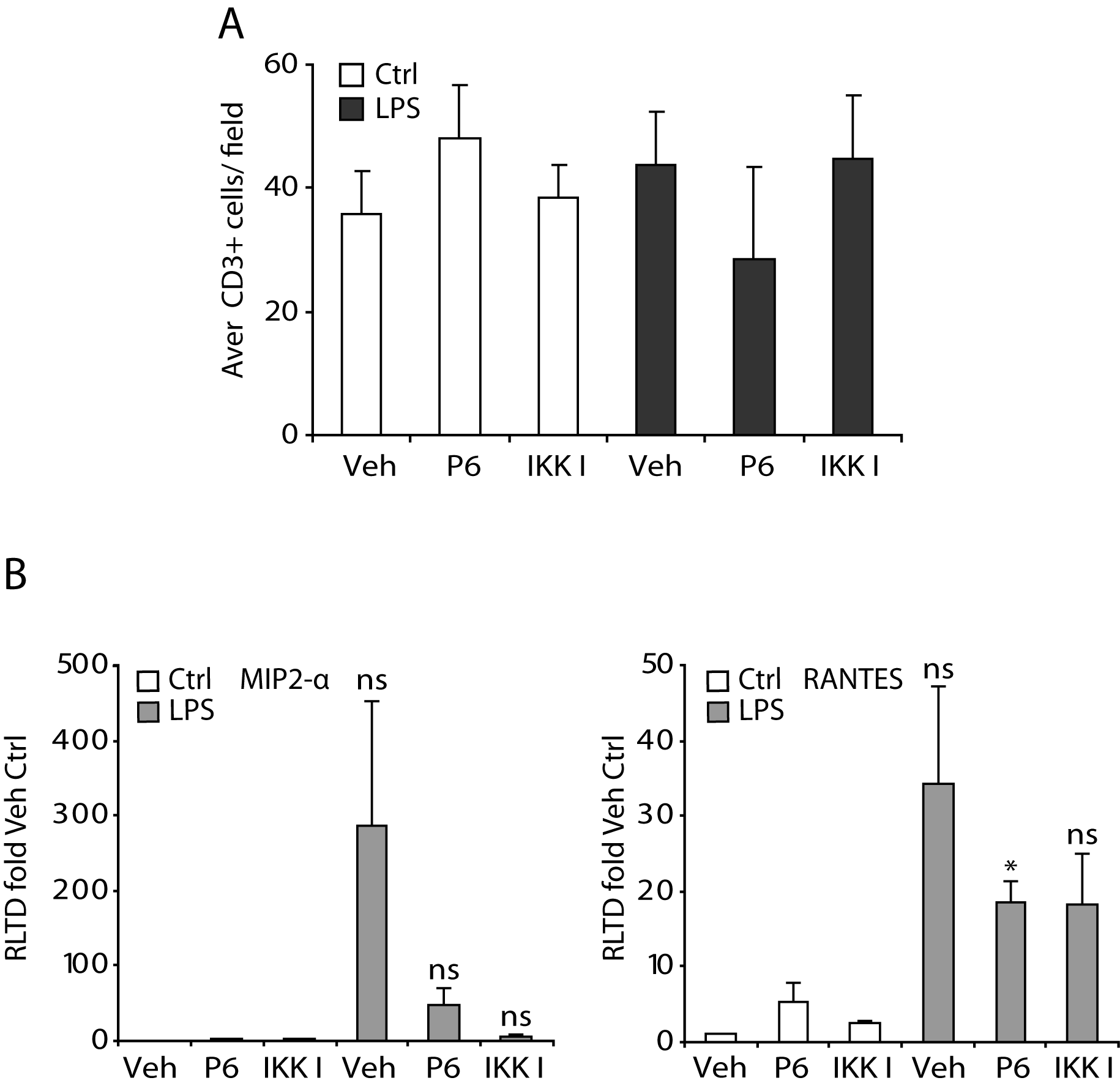

Supplement: Supplementary file 1 [file hep0057-0817-sd1.tif]

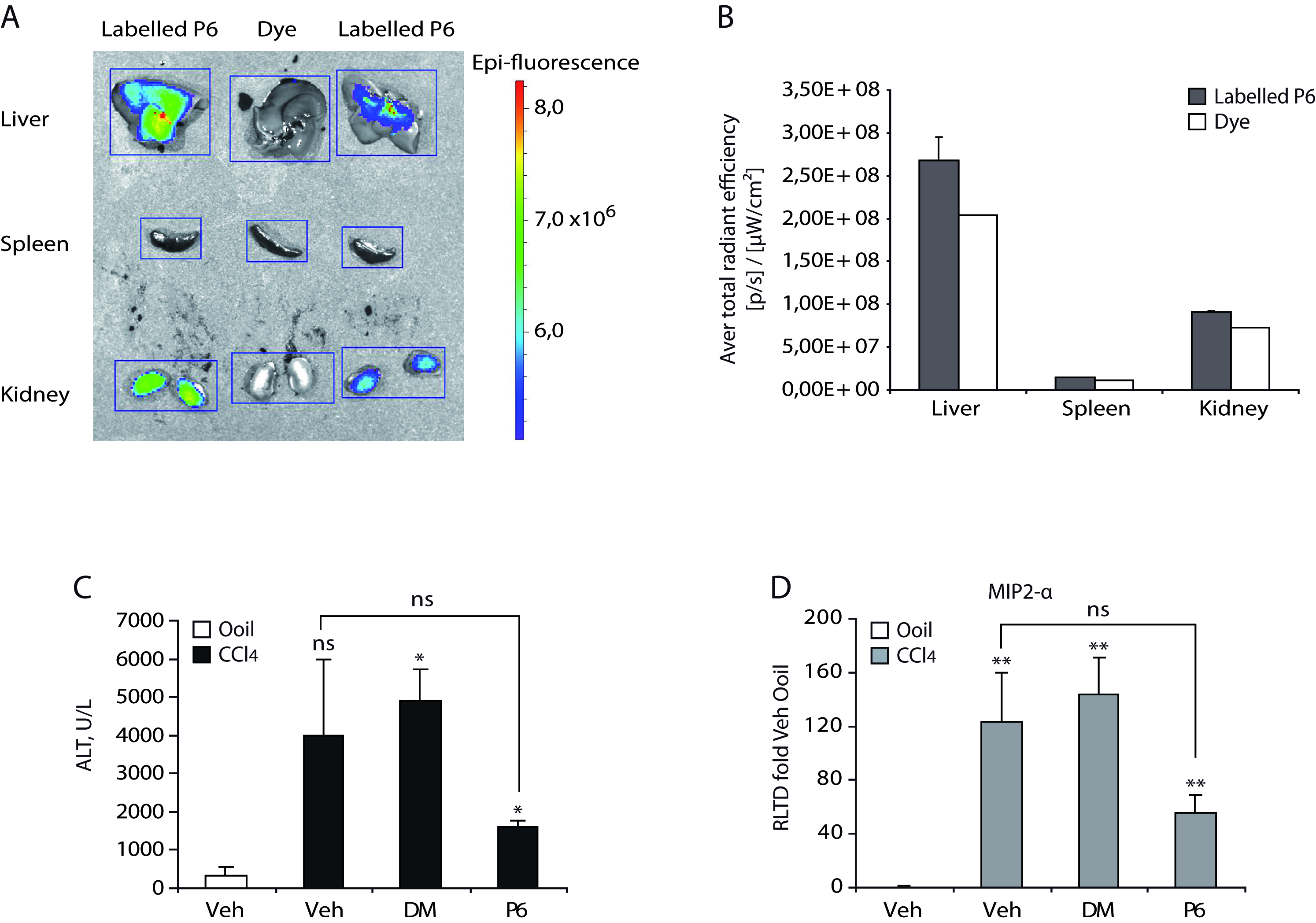

Supplement: Supplementary file 2 [file hep0057-0817-sd2.tif]

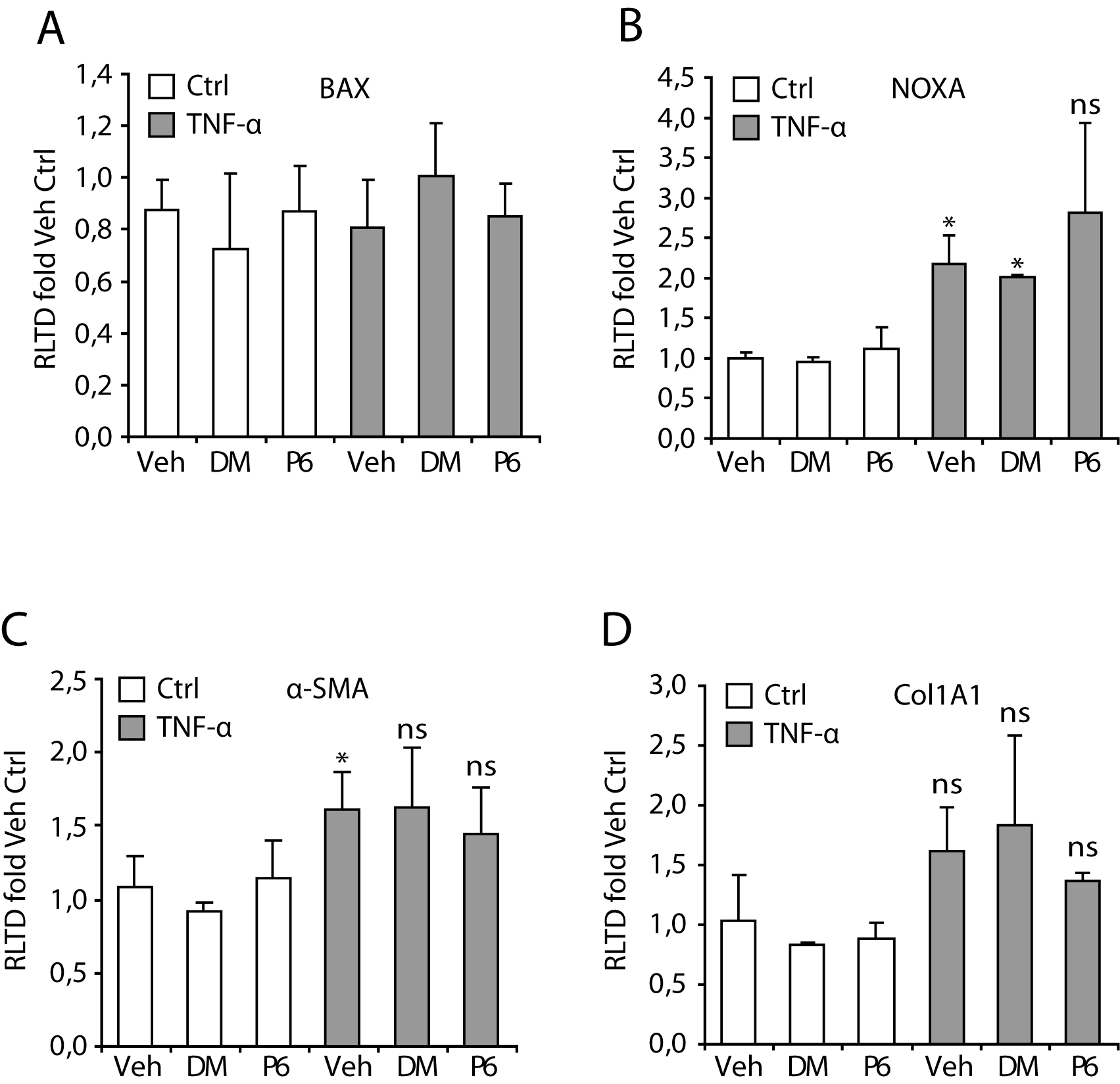

Supplement: Supplementary file 3 [file hep0057-0817-sd3.tif]

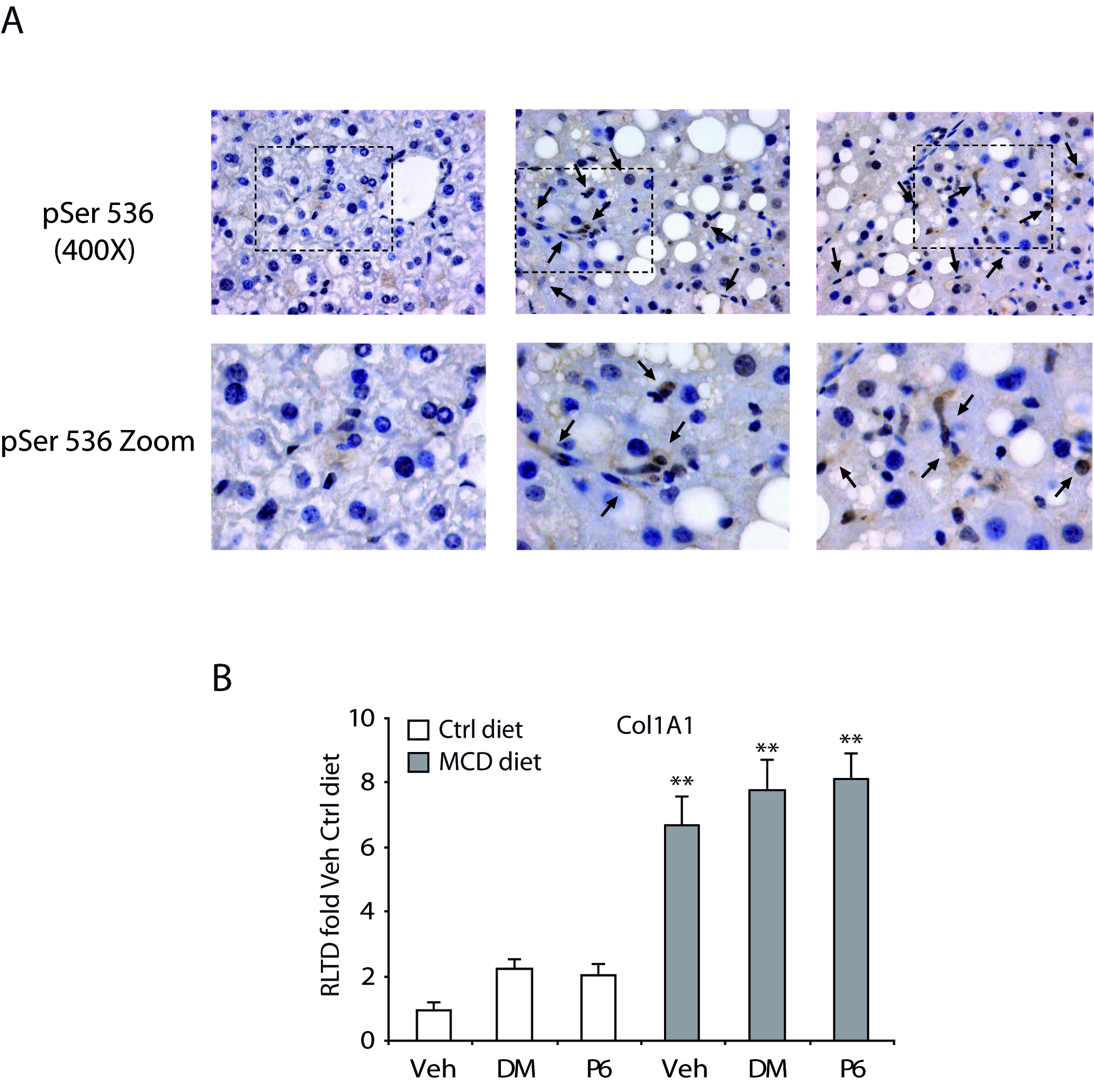

Supplement: Supplementary file 4 [file hep0057-0817-sd4.tif]

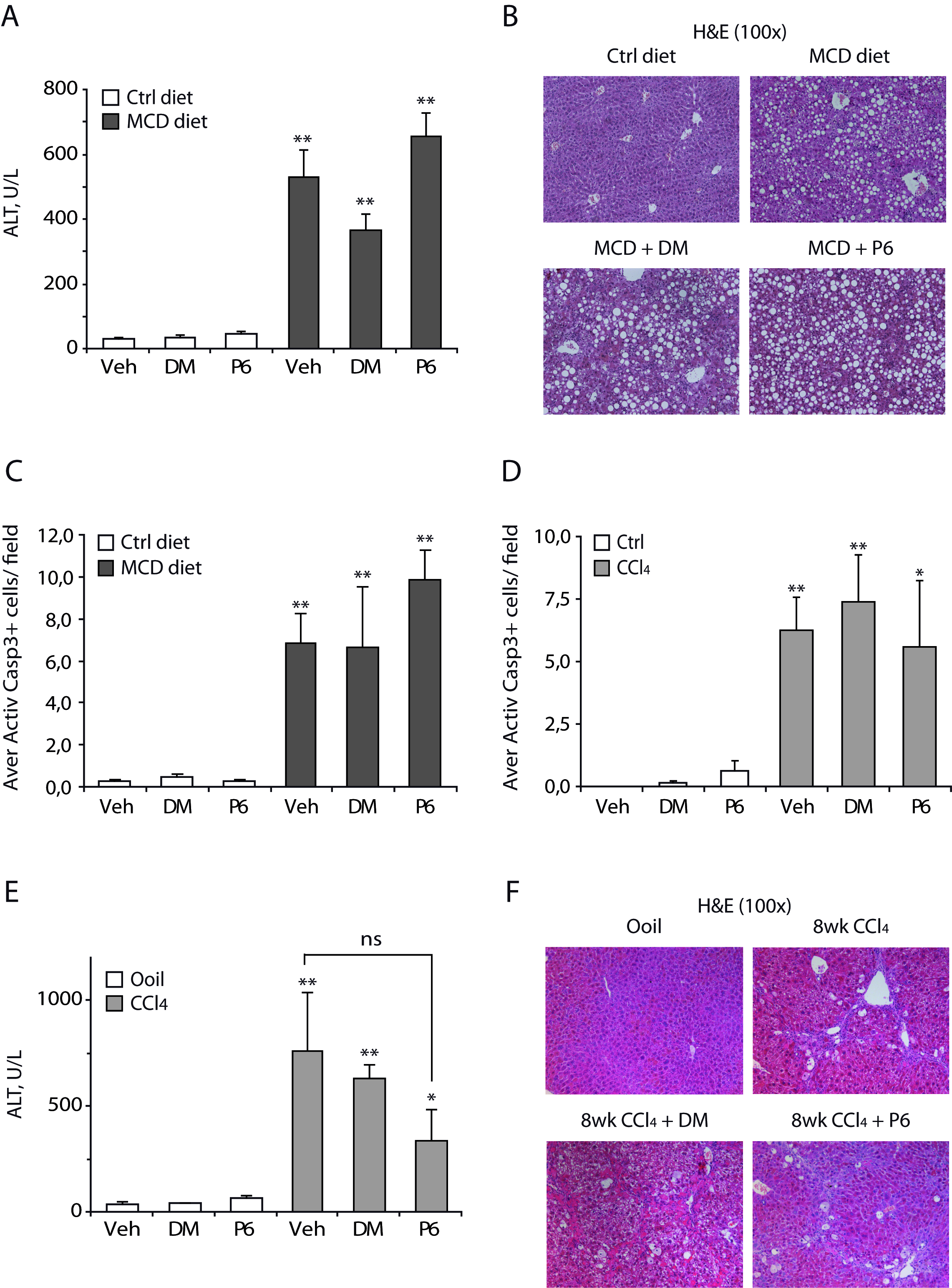

Supplement: Supplementary file 5 [file hep0057-0817-sd5.tif]

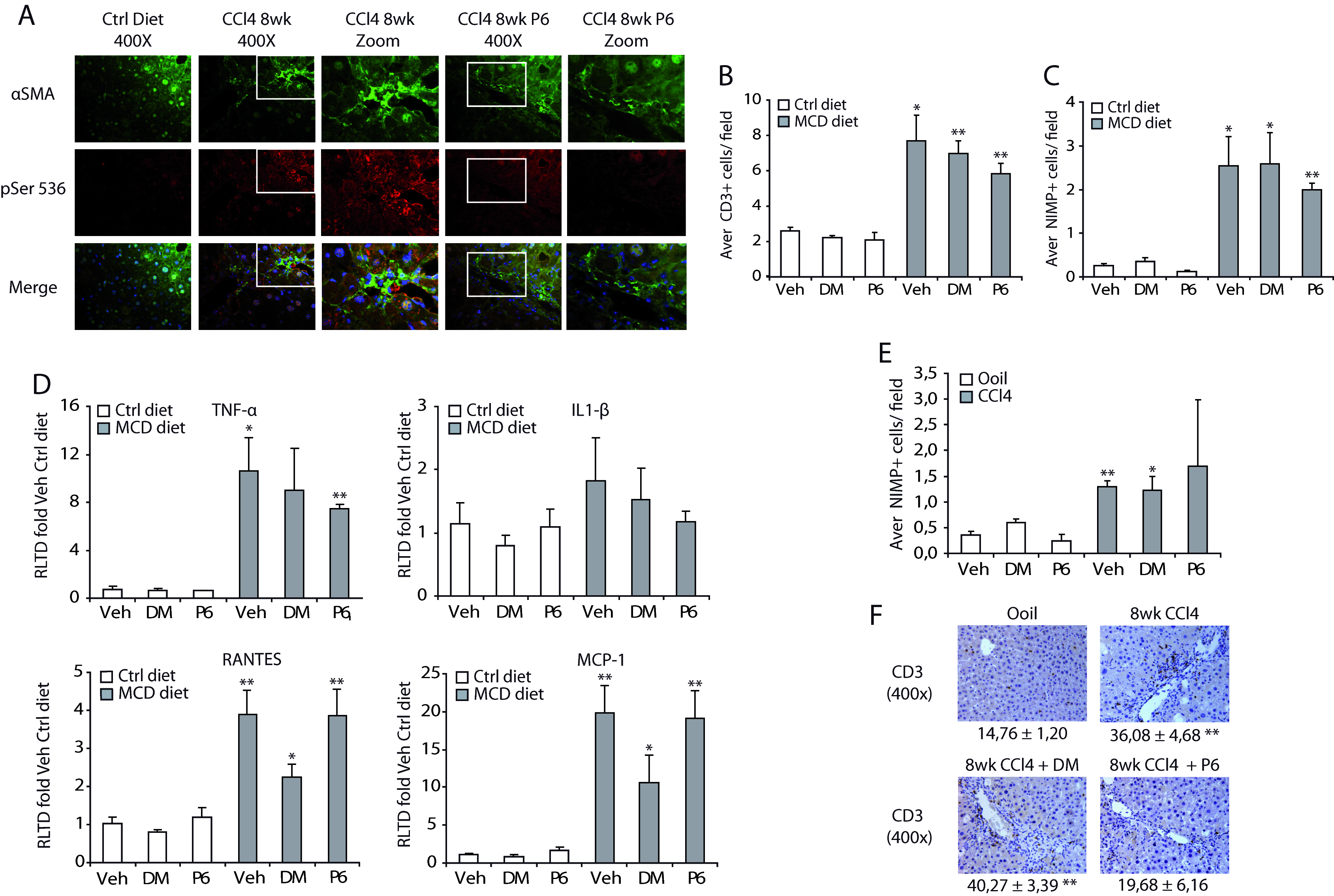

Supplement: Supplementary file 6 [file hep0057-0817-sd6.tif]

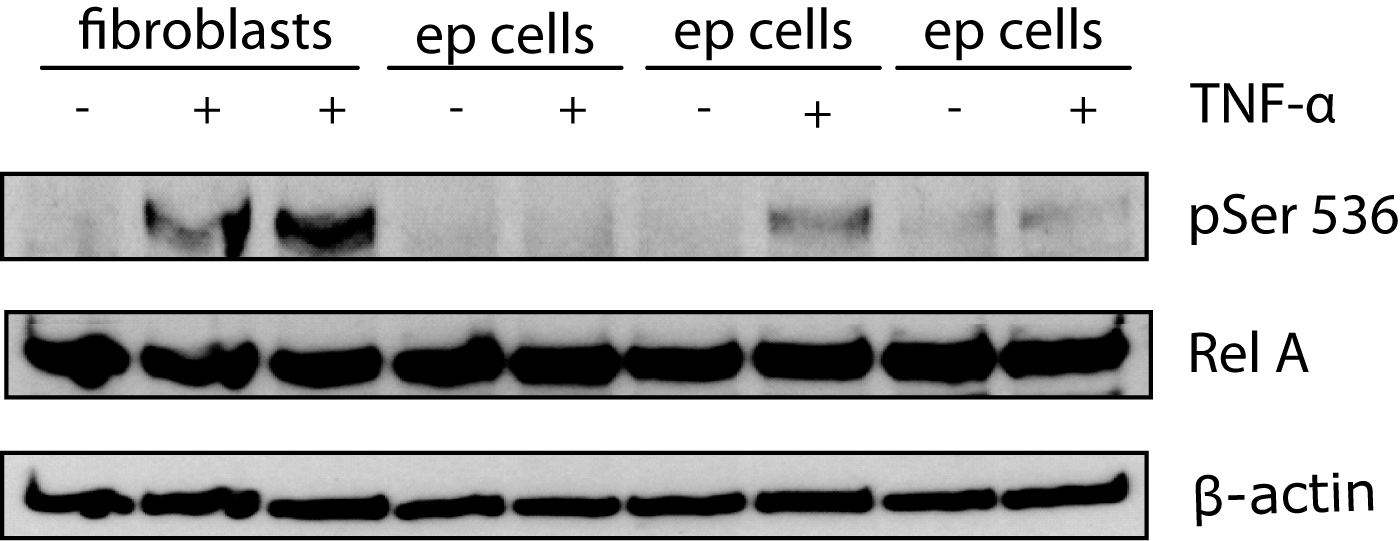

Supplement: Supplementary file 7 [file hep0057-0817-sd7.tif]
