# Supplementary material for: Inhibition of RelA-Ser536 Phosphorylation by a Competing Peptide Reduces Mouse Liver Fibrosis Without Blocking the Innate Immune Response
Source: Hepatology. 2013 Jan 8;57(2):817–28. doi: 10.1002/hep.26068 (PMC3807604; doi:10.1002/hep.26068)
Supplement: Supplementary file 8 [file hep0057-0817-sd8.doc]

**Supplementary methods:**

**Reagents.** Human TNF-α (PreProtech) was used at 50ng/mL unless stated. Recombinant IKKα and IKKβ were from Millipore. Recombinant Protein G Beads were from Generon. Methionine Choline deficient and Control diet were from Research Diets. Avidin/Biotin blocking kit, Citric acid antigen unmasking solution, Vectastain Elite ABC Reagent, DAB peroxidase substrate kit and Vectashield-DAPI mounting medium were from Vector Laboratories. Unless otherwise reported all reagents were from Sigma-Aldrich.

**Immunofluorescence.** Dual fluorescence was performed in formalin fixed liver. Sections were deparaffinised and citrate saline antigen retrieval was performed. Tissues were permeabilised with 0.25% Triton X-100 and blocked with 3% BSA. α-SMA-FITC and RelA-P-Ser^536^ (Cell Signalling) were incubated in 1% BSA overnight at 4°C. After PBS washing, biotinylated secondary antibody and Streptavidin-594 tertiary antibody were sequentially added. Sections were mounted with Vectashield mounting medium. Human HM up to passage 4 were cultured on coverslips in 0.5% FBS DMEM and treated with TNF-α 50ng/mL for 10 minutes. Coverslips were fixed with 3.7% formaldehyde for 10 minutes. Immunocytochemistry was performed as previously described (1).

**SDS-PAGE and immunoblotting.** Cells were lysed in RIPA buffer with protease and phosphotase inhibitors. Proteins were fractionated by SDS-PAGE, transferred to nitrocellulose then blocked with TBS/Tween 20 (0.1%, T-TBS) containing 5% nonfat milk or BSA before overnight incubation with primary antibodies anti-RelA-P-Ser^536^ or anti-α-Tubulin (Cell Signaling 1:1000), anti-Lamin B1 (Abcam, 1:1000). Membranes were washed in T-TBS and incubated with either anti-rabbit or anti-mouse HRP conjugated for 2 hours. Blots were washed and antigen detected by ECL (Thermo Scientific). Conditions for RelA, β–actin, IκBα, IKKα/β and P-IKKα/β at serine 180/1 have been previously described (9).

**Nuclear and cytosolic extracts preparation.** LX2 cells were lysate with 10 mM Hepes pH 7.9, containing 10 mM KCl, 0.1 mM EDTA, 0.1 mM EGTA, 1 mM DTT, 0.5% Igepal. Cells were incubated on ice for 15 minutes, vigorously vortex and centrifuge at 13000rpm at 4ºC for 30 seconds. Supernatant or cytosol was frozen down at -80ºC. Pellet or nuclei was resuspended and incubated for 15 minutes on ice in 20 mM Hepes pH 7.9, 0.4 M NaCl, 1 mM EDTA, 1 mM EGTA, 1 mM DTT. After centrifuged at 13000rpm 4ºC for 5 minutes supernatant or nuclear extracts were stored at -80ºC.

**In-vivo peptide tracking by IVIS imaging**. P6 peptide was labelled using DyLight 800 Antibody Labeling Kit (Thermo). Peptide at 10mg/kg or empty probe was injected IP into 8-10 weeks C57BL/6 mice. Animals were scanned using 745nm excitation and 800nm emission wavelengths with an IVIS Spectrum CT (Caliper Life Sciences) at 30 minutes and 2, 3, 4, 6, 24 hours. Animals were scanned before injection and the background was subtracted. Data was analysed using Living Image 4.2 software, regions of interest (ROI) were drawn and the average of the Total Radiant Efficiency [p/s] / [µW/cm²] was calculated per ROI versus the liver ROI.

**Reference**

1. Moles A, Tarrats N, Fernandez-Checa JC, Mari M. Cathepsins B and D drive hepatic stellate cell proliferation and promote their fibrogenic potential. Hepatology 2009;49:1297-1307.
