# Supplementary material for: Inhibition of RelA-Ser536 Phosphorylation by a Competing Peptide Reduces Mouse Liver Fibrosis Without Blocking the Innate Immune Response
Source: Hepatology. 2013 Jan 8;57(2):817–28. doi: 10.1002/hep.26068 (PMC3807604; doi:10.1002/hep.26068)
Supplement: Supplementary file 9 [file hep0057-0817-sd9.doc]

| Peptide Name | Aminoacid sequence |
| --- | --- |
| P6 (active peptide) | Acetyl- DRQIKIWFQNRRMKWKKNGLLSGDEDFSS-COOH |
| DM (control peptide) | Acetyl- DRQIKIWFQNRRMKWKKNGLL**A**GDEDFS**A**-COOH |
| M (control peptide) | Acetyl- DRQIKIWFQNRRMKWKKNGLLSGDEDFS**A**-COOH |

**Supplemental table 1: Peptide sequences**

Peptides contain the penetrating homeodomain from Antennapedia (DRQIKIWFQNRRMKWKK) plus the peptide span aminoacids from 525-536 of RelA.

DM and M are control peptides. In DM serine 529 and 536 are replaced with non-phosphorylatable Alanine residues. M contains serine 529 but Serine 536 is substituted to Alanine.
