# Supplementary material for: Inhibition of RelA-Ser536 Phosphorylation by a Competing Peptide Reduces Mouse Liver Fibrosis Without Blocking the Innate Immune Response
Source: Hepatology. 2013 Jan 8;57(2):817–28. doi: 10.1002/hep.26068 (PMC3807604; doi:10.1002/hep.26068)
Supplement: Supplementary file 10 [file hep0057-0817-sd10.doc]

**Supplemental table 3: Human** primer sequences

| **Gene ( GenBank Accession)** | **Primer sequence** |
| --- | --- |
| GAPDH (NM_002046) | Fw: 5’-GAAGGTGAAGGTCGGAGTC -3’ |
|  | Rv: 5’-GAAGATGGTGATGGGATTTC -3’ |
| α-SMA (NM_001613) | Fw: 5’-GCGTGGCTATTCCTTCGTTACT-3’ |
|  | Rv: 5’-CCGATGAAGGATGGCTGGAACA-3’ |
| Collagen 1A1 (NM_000088) | Fw: 5’-CAAGAGGAAGGCCAAGTC-3’ |
|  | Rv: 5’-CGTTGTCGCAGACGCAGAT-3’ |
| NOXA (NM_021127) | Fw: 5’-TGGAAGTCGAGTGTGCTACTCAA-3’ |
|  | Rv: 5’-TTTCTGCCGGAAGTTCAGTTTGTCTCCAA -3’ |
| DR5 (NM_003842) | Fw: 5’-GGTGAAGTGGAGCTAAGTCCC-3’ |
|  | Rv: 5’-CACTGTGCTTTGTACCTGATTCT-3’ |
| A20 (NM_024309) | Fw: 5’-CACGAGCCCGAGCTGATGAGG-3’ |
|  | Rv: 5’-CTTCCCCTTGCTCGTCACTG-3’ |
| GADD45-β (NM_015675) | Fw: 5’-GCATACGAGAGACTTGGTTGA -3’ |
|  | Rv: 5’-GCTTCCCATCTCGCTCTCAGT-3’ |
| BAX ( NM_138761) | Fw: 5’-TAACATGGAGCTGCAGAG-3’ |
|  | Rv: 5’-CAGTTTGCTGGCAAAGTA-3’ |
